# Naldemedine and Magnesium Oxide as First-Line Medications for Opioid-Induced Constipation: A Comparative Database Study in Japanese Patients With Cancer Pain

**DOI:** 10.7759/cureus.55925

**Published:** 2024-03-10

**Authors:** Takaomi Kessoku, Takahiro Higashibata, Yasuhide Morioka, Noriyuki Naya, Yuichi Koretaka, Yasushi Ichikawa, Takayuki Hisanaga, Atsushi Nakajima

**Affiliations:** 1 Department of Palliative Medicine, International University of Health and Welfare Narita Hospital, Narita, JPN; 2 Department of Gastroenterology, International University of Health and Welfare School of Medicine, Narita, JPN; 3 Department of Gastroenterology and Hepatology, Yokohama City University School of Medicine, Yokohama, JPN; 4 Department of Palliative and Supportive Care, University of Tsukuba Hospital, Tsukuba, JPN; 5 Department of Medical Affairs, Shionogi & Co. Ltd., Osaka, JPN; 6 Department of Data Science, Shionogi & Co. Ltd., Osaka, JPN; 7 Department of Oncology, Yokohama City University School of Medicine, Yokohama, JPN; 8 Department of Palliative Medicine, Tsukuba Medical Center Hospital, Tsukuba, JPN

**Keywords:** opioids, naldemedine, magnesium oxide, japan, database, constipation, cancer, analgesics

## Abstract

Introduction

Naldemedine and magnesium oxide are common first-line early laxative medications used in the real-world scenario in Japan, for patients with cancer pain who receive opioid prescriptions, as per a nationwide hospital claims database study. However, the real-world prescription patterns and associated outcomes are unknown.

Methods

In this retrospective, cohort study using the Medical Data Vision (MDV) database (January 2018 to December 2020), data were collected from eligible patients (who had a long-term prescription of strong opioids, for >30 days) in Japan with naldemedine or magnesium oxide as the first-line laxative prescription, for a long-term opioid prescription for cancer pain with ≥6 months post-opioid observation period. A laxative prescription within three days after the opioid prescription date was termed an "early" prescription. The composite incidence of dose increase or addition/change of laxatives at three months after the start of the opioid prescription was the primary endpoint after adjusting baseline characteristics between the treatment arms by propensity score matching.

Results

After propensity score matching, 1717 and 544 patients who were prescribed naldemedine and magnesium oxide each were included in the early prescription and non-early prescription groups, respectively. Even after matching, the incidence of death was not adjusted enough and was significantly higher in the naldemedine arm than in the magnesium oxide arm in the non-early group but comparable in the early group. The incidence of addition, change, or dose increase was significantly higher in the naldemedine arm than in the magnesium oxide arm of the early prescription group (hazard ratio (95% confidence interval), 1.08 (1.00, 1.17); *p*=0.0402); the incidence was comparable between the arms of the non-early group.

Conclusion

These findings may provide valuable insights into real-world clinical treatment patterns and preliminary evidence for the selection of first-line medications to mitigate opioid-induced constipation in Japanese patients with cancer pain.

## Introduction

Although opioids are commonly used to treat cancer pain because of their potent analgesic activity, their use is associated with adverse effects, such as sedation, physical dependence, respiratory depression, and gastrointestinal side effects [1]. The most pronounced gastrointestinal effect is opioid-induced constipation (OIC), primarily mediated by μ-opioid receptors (MORs) in the enteric system [2,3]. The chronic nature of OIC negatively affects pain management [4,5] and patient's health-related quality of life (HRQoL) [1,6,7].

As for the treatment of OIC, the American Gastroenterological Association (AGA) Institute Guideline on the Medical Management of OIC and the Japanese Society of Palliative Medicine recommend traditional laxatives as first-line agents [3,8]. However, approximately half the patients with OIC do not achieve the desired improvement with traditional laxatives, presumably because they do not target the underlying cause of OIC, i.e., MORs in the enteric system [9]. Accordingly, in patients with traditional laxative refractory OIC, the AGA guideline recommends escalating the therapy with recently developed agents, such as peripherally acting μ-opioid receptor antagonists (PAMORAs), including a strong recommendation for naldemedine which blocks MORs in the gut, thereby effectively reversing OIC without affecting central analgesia [3].

An observational study in Japan demonstrated that prophylactic intake of laxatives, including magnesium oxide, an osmotic laxative, at the start of opioid therapy attenuated OIC [10]. However, magnesium oxide has been used mostly based on empirical knowledge in Japan [11]. The efficacy/effectiveness and safety of naldemedine for the treatment of OIC in patients with cancer pain are amply documented in prospective, randomized controlled trials and observational, real-world studies under different clinical settings in Japan, including post-marketing surveillance, retrospective chart review, etc. [12-14]. The efficacy/effectiveness is explained by the mechanism of naldemedine, which targets the underlying cause of OIC. Additionally, preliminary studies have suggested that naldemedine may have a beneficial effect on opioid-induced nausea and vomiting (OINV) [15,16]. Some clinical studies, including prospective, randomized, double-blind, placebo-controlled trials for chronic constipation; a retrospective, cohort study for OIC; and a randomized controlled trial comparing magnesium oxide to naldemedine, were reported [17-19]. In an open-label, randomized controlled trial, naldemedine prevented deterioration in constipation-specific HRQoL and complete spontaneous bowel movement rate compared with magnesium oxide in patients with cancer who were on opioid therapy [19]. However, no study has yet assessed the treatment status and associated outcomes when naldemedine and magnesium oxide are prescribed as first-line medications for the treatment of OIC in real-world clinical settings. In real-world clinical settings, laxatives, including naldemedine, were found to tend to be prescribed early (in three days) after initiating opioids [20]. Moreover, the number of prescription days and usage for osmotic laxatives such as magnesium oxide and PAMORAs such as naldemedine were similar and longer than those for other laxatives [20]. Herein, we report findings from an epidemiological database study on naldemedine and magnesium oxide as the first-line medications for OIC patients with cancer pain using a Medical Data Vision (MDV) database.

## Materials and methods

Study design

This was a retrospective, cohort study based on an administrative health database with medical data from real-world opioid-prescribed patients.

Data source

We used the MDV database, a medical information database constructed based on the anonymized diagnostic procedure combination data provided by Medical Data Vision Co., Ltd. for this study [21]. The MDV database captured hospitalization data along with the subsequent outpatient treatment at the hospital, along with patient data of all ages without bias in the age composition. It included patient data, disease data coded according to Japanese Claims Codes and International Statistical Classification of Diseases and Related Health Problems (ICD-10) codes, degree of nursing care, activities of daily living (ADL) scores, drug codes including chemotherapy, and laboratory data. As of January 2020, MDV contained data from 397 acute medical institutions, including 190 hospitals that partake in cancer treatment.

Study period

The study period for this retrospective study was from January 2018 to December 2020.

Study population

Patients with a diagnosis of a malignant tumor (corresponding ICD-10 codes are reported in Appendix A) and a long-term prescription of opioids (>30 days) after diagnosis of the oldest malignant tumor between January 2018 and June 2020 were eligible. Oral opioids, viz., oxycodone, tapentadol, hydromorphone, morphine, and topical fentanyl, were permitted as starting opioids. Patients who could be observed for at least six months from the start of opioid prescription (i.e., those who had opioid prescription start date before July 1, 2020) and those with naldemedine or magnesium oxide prescribed as the first-line laxative after opioid prescription were included. Exclusion criteria were patients with an opioid prescription within seven days before a long-term opioid prescription (excluding rescue prescriptions reported in Appendix B) and prescriptions for multiple laxative medications on the same day or laxatives other than naldemedine or magnesium oxide as the first-line medication.

Analysis population

The first-line laxative prescribed after the index date, the date of long-term prescription of opioids, was defined as the first-line medication naldemedine as the intervention arm or magnesium oxide as the control arm. Eligible patients were divided into groups with early and non-early prescriptions for naldemedine or magnesium oxide after the index date. An early prescription was defined as a prescription for naldemedine or magnesium oxide within three days after the index date, whereas a prescription for naldemedine or magnesium oxide on or after the fourth day after the index date was defined as a non-early prescription. In eligible patients, the observation period was from the index date to the date of death or the date of last medical records available within the study period, whichever was longer.

Outcomes

The primary endpoint was a composite incidence of dose increase or addition/change of laxatives three months after the start of the opioid prescription. The secondary endpoints included treatment for constipation (increased dose, addition/change of laxatives, and incidence of constipation treatment including stool extraction, enema, etc.), impact on opioid use (amount used and change), impact on management for other opioid-induced side effects (nausea treatment: use of antiemetics), and general condition (total medical expenses and number of deaths). The opioid dose was converted to a morphine equivalent dose (Appendix C).

Statistical analysis

For the patient background, categorical variables were reported using frequency and percentages, while continuous variables were reported as mean and standard deviation (SD). Correspondingly, chi-squared tests and t-tests were performed before and after propensity score matching as two-sided tests at a significance level of p<0.05 for assessing the inter‑group differences between naldemedine and magnesium oxide arms with early and non-early prescriptions.

Data from naldemedine and magnesium oxide arms were compared after adjusting their baseline characteristics by propensity score matching. The propensity score was calculated by logistic regression analysis with the background information of each case as a covariate, and the confounding of patient background factors was adjusted using matching. The following covariates used for propensity score calculation were common for patient groups with early and non-early prescriptions: age, gender, type of cancer, opioid prescription category (outpatient/inpatient) on the index date, time to start of laxative prescription, type of opioid on the index date, and presence or absence of peritoneal dissemination (Appendix D). The opioid dose was not included as a covariate for propensity score calculation, as OIC is not dependent on the opioid dose. Based on the logit value of the calculated propensity score, 1:1 matching between naldemedine and magnesium oxide arms was performed with the following settings: sampling without replacement, nearest neighbor matching, and caliper settings at 0.25 times the standard deviation of the propensity score logit value.

Each primary and secondary endpoint analysis was conducted on a population with matching patient backgrounds. For each categorical endpoint, the hazard ratios (HRs, with 95% confidence interval (CI)) of event occurrence in the naldemedine versus the magnesium oxide arm (as a referent) were calculated in the groups with and without early prescription, and a log-rank test was performed. For continuous endpoints, a t-test was performed when a normal distribution could be assumed, whereas Mann-Whitney's U test was performed when a normal distribution could not be assumed. SAS v9.4 software (SAS Institute Inc., Cary, North Carolina, United States) was used for all statistical comparisons.

Ethical considerations

This study was based on anonymized structured data, not subject to privacy laws. So, informed consent from patients and institutional review board/independent ethics committee approval were not required according to the Ethical Guidelines on Medical Research for Human Beings [22].

## Results

**Figure 1:**
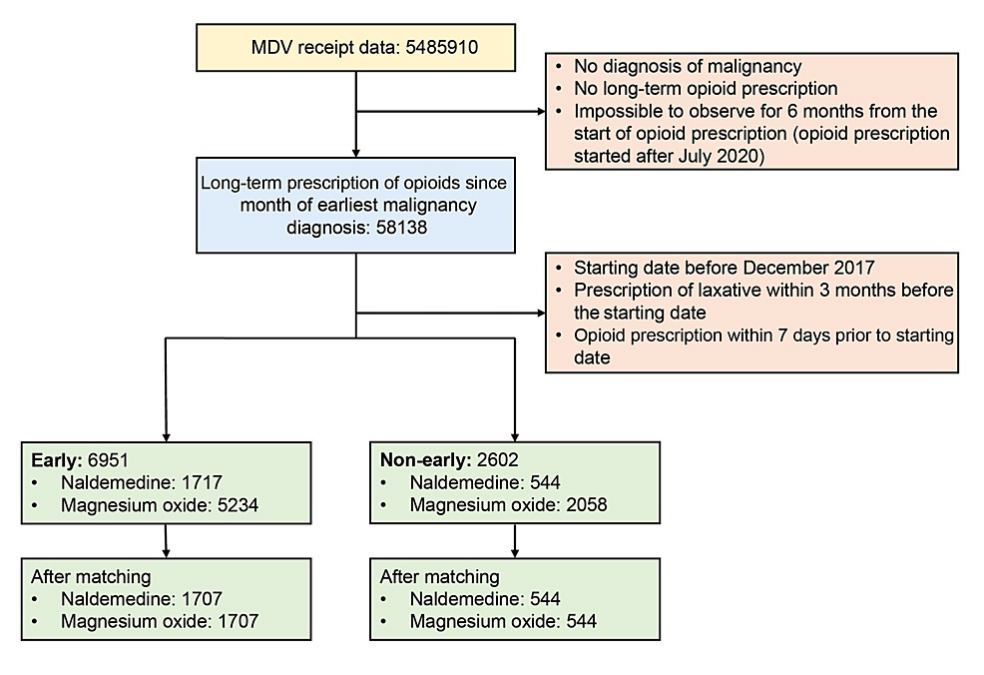
Patient disposition MDV: Medical Data Vision

In the early prescription group (n=6951), 1717 (24.7%) patients were prescribed naldemedine, whereas 5234 (75.3%) patients were prescribed magnesium oxide (Figure 1).

**Figure 2:**
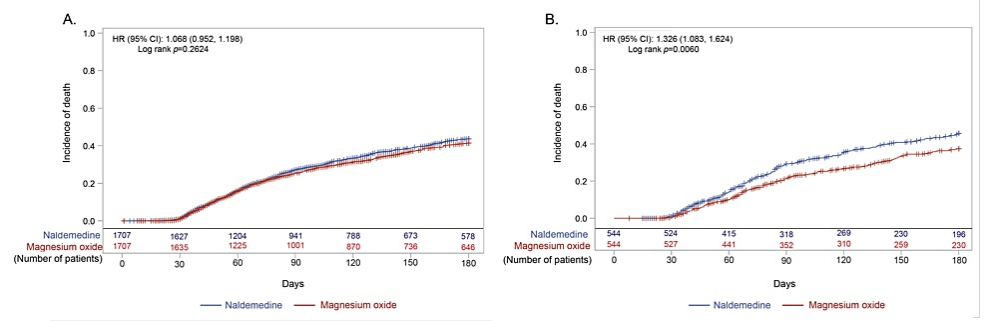
Incidence of death after the start of the opioid prescription in (A) early and (B) non-early prescription groups CI: confidence interval; HR: hazard ratio

In the non-early prescription group (n=2602), 544 (20.9%) and 2058 (79.1%) patients were prescribed naldemedine and magnesium oxide, respectively (Figure 1). Overall, 1717 matched patients in the early prescription group and 544 matched patients in the non-early prescription group were included in the study analysis. Patient background characteristics after matching were comparable between the naldemedine and magnesium oxide arms for both early and non-early prescription groups (Table 1 and Table 2). However, even after matching, the incidence of death was significantly higher in the naldemedine arm than in the magnesium oxide arm of the non-early prescription group, but comparable between the naldemedine and magnesium oxide arms of the early prescription group (Figure 2).

**Table 1:** Baseline characteristics of patients with early prescriptions Data are presented as n (%) unless stated otherwise. SD: standard deviation

	Before matching		After matching			
	Naldemedine (n=1717)	Magnesium oxide (n=5234)	Naldemedine (n=1707)	Magnesium oxide (n=1707)	P-value	Standard difference
Mean age (SD); years	68.2 (12.4)	68.4 (12.1)	68.2 (12.4)	68.4 (12.4)	0.7041	-0.0131
Sex					0.3699	-0.0308
Male	972 (56.6)	3053 (58.3)	971 (56.9)	945 (55.4)	-	-
Female	745 (43.4)	2181 (41.7)	736 (43.1)	762 (44.6)	-	-
Cancer type						
Head and neck	39 (2.3)	152 (2.9)	39 (2.3)	25 (1.5)	0.0773	0.0517
Digestive tract	182 (10.6)	535 (10.2)	180 (10.5)	171 (10.0)	0.6120	0.0173
Liver, gall, pancreas	214 (12.5)	524 (10.0)	213 (12.5)	217 (12.7)	0.8365	-0.0074
Lungs	206 (12.0)	611 (11.7)	206 (12.1)	217 (12.7)	0.5677	-0.0199
Mammary gland	45 (2.6)	168 (3.2)	45 (2.6)	50 (2.9)	0.6029	-0.0174
Gynecologic cancer	59 (3.4)	108 (2.1)	53 (3.1)	57 (3.3)	0.6983	-0.0143
Renal and urological	74 (4.3)	213 (4.1)	74 (4.3)	74 (4.3)	1.0000	0.0000
Blood	32 (1.9)	205 (3.9)	32 (1.9)	32 (1.9)	1.0000	0.0000
Others	100 (5.8)	308 (5.9)	100 (5.9)	80 (4.7)	0.1256	0.0499
Overlap	766 (44.6)	2410 (46.0)	765 (44.8)	784 (45.9)	0.5137	-0.0224
Oral opioids						
Oxycodone hydrochloride hydrate	1104 (64.3)	3955 (75.6)	1104 (64.7)	1140 (66.8)	0.1942	-0.0463
Tapentadol hydrochloride	62 (3.6)	149 (2.8)	61 (3.6)	55 (3.2)	0.5709	0.0199
Hydromorphone hydrochloride	342 (19.9)	374 (7.1)	333 (19.5)	337 (19.7)	0.8631	-0.0070
Morphine	80 (4.7)	276 (5.3)	80 (4.7)	64 (3.7)	0.1731	0.0431
Topical opioids						
Fentanyl	140 (8.2)	506 (9.7)	140 (8.2)	123 (7.2)	0.2752	0.0350
Prescription category					0.8098	-0.0082
Outpatient	943 (54.9)	2774 (53.0)	936 (54.8)	929 (54.4)	-	-
Hospitalization	774 (45.1)	2460 (47.0)	771 (45.2)	778 (45.6)	-	-
Peritoneal metastasis	172 (10.0)	380 (7.3)	167 (9.8)	150 (8.8)	0.3161	0.0355

**Table 2:** Baseline characteristics of patients with non-early prescriptions Data are presented as n (%) unless stated otherwise. SD: standard deviation

	Before matching		After matching			
	Naldemedine (n=544)	Magnesium oxide (n=2058)	Naldemedine (n=544)	Magnesium oxide (n=544)	P-value	Standard difference
Mean age (SD); years	68.1 (12.0)	67.9 (12.1)	68.1 (12.0)	68.8 (11.8)	0.3662	-0.0539
Sex					0.6255	0.0297
Male	298 (54.8)	1204 (58.5)	298 (54.8)	306 (56.3)	-	-
Female	246 (45.2)	854 (41.5)	246 (45.2)	238 (43.8)	-	-
Cancer type						
Head and neck	9 (1.7)	45 (2.2)	9 (1.7)	9 (1.7)	1.0000	0.0000
Digestive tract	86 (15.8)	278 (13.5)	86 (15.8)	77 (14.2)	0.4446	0.0468
Liver, gall, pancreas	65 (11.9)	221 (10.7)	65 (11.9)	68 (12.5)	0.7813	-0.0174
Lungs	49 (9.0)	221 (10.7)	49 (9.0)	60 (11.0)	0.2667	-0.0678
Mammary gland	9 (1.7)	70 (3.4)	9 (1.7)	7 (1.3)	0.6145	0.0234
Gynecologic cancer	24 (4.4)	47 (2.3)	24 (4.4)	21 (3.9)	0.6478	0.0307
Renal and urological	29 (5.3)	93 (4.5)	29 (5.3)	26 (4.8)	0.6780	0.0255
Blood	11 (2.0)	87 (4.2)	11 (2.0)	9 (1.7)	0.6517	0.0212
Others	29 (5.3)	92 (4.5)	29 (5.3)	25 (4.6)	0.5766	0.0340
Overlap	233 (42.8)	904 (43.9)	233 (42.8)	242 (44.5)	0.5822	-0.0334
Oral opioids						
Oxycodone hydrochloride hydrate	338 (62.1)	1367 (66.4)	338 (62.1)	328 (60.3)	0.5338	0.0384
Tapentadol hydrochloride	37 (6.8)	86 (4.2)	37 (6.8)	38 (7.0)	0.9047	-0.0081
Hydromorphone hydrochloride	78 (14.3)	172 (8.4)	78 (14.3)	82 (15.1)	0.7320	-0.0233
Morphine	23 (4.2)	82 (4.0)	23 (4.2)	22 (4.0)	0.879	0.0093
Topical opioids						
Fentanyl	70 (12.9)	355 (17.2)	70 (12.9)	75 (13.8)	0.6556	-0.0257
Prescription category					0.7926	0.0157
Outpatient	375 (68.9)	1372 (66.7)	375 (68.9)	379 (69.7)	-	-
Hospitalization	169 (31.1)	686 (33.3)	169 (31.1)	165 (30.3)	-	-
Time until the start of laxative prescription					0.8117	-
~2 weeks	251 (46.1)	872 (42.4)	251 (46.1)	250 (46.0)	-	0.0037
>2 weeks to 1 month	138 (25.4)	501 (24.3)	138 (25.4)	148 (27.2)	-	-0.0425
>1 to 2 months	64 (11.8)	280 (13.6)	64 (11.8)	53 (9.7)	-	0.0608
>2 to 3 months	27 (5.0)	132 (6.4)	27 (5.0)	25 (4.6)	-	0.0159
>3 months	64 (11.8)	273 (13.3)	64 (11.8)	68 (12.5)	-	-0.0222
Peritoneal metastasis	52 (9.6)	204 (9.9)	52 (9.6)	38 (7.0)	0.1234	0.0868

**Figure 3:**
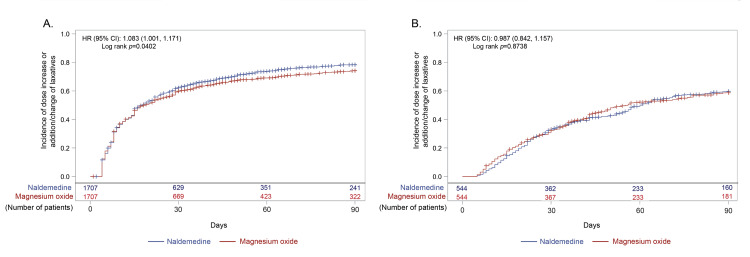
Incidence of dose increase or addition/change of laxatives after the start of the opioid prescription in (A) early and (B) non-early prescription groups CI: confidence interval; HR: hazard ratio

**Figure 4:**
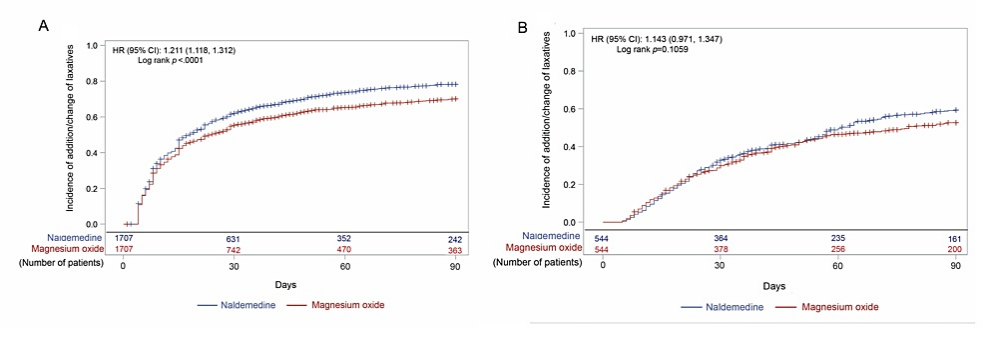
Incidence of addition/change of laxatives after the start of the opioid prescription in (A) early and (B) non-early prescription groups CI: confidence interval; HR: hazard ratio

**Figure 5:**
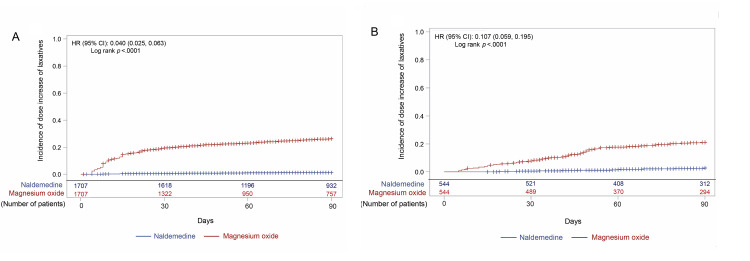
Incidence of dose increase of laxatives after the start of the opioid prescription in (A) early and (B) non-early prescription groups CI: confidence interval; HR: hazard ratio

About 80% and 60% of patients received addition, change, or dose increase of laxatives after opioid initiation in the early prescription and non-early prescription groups, respectively (Figure 3(A), Figure 3(B)). The incidence of addition, change, or dose increase was slightly but significantly higher in the naldemedine arm than in the magnesium oxide arm of the early prescription group (HR (95% CI), 1.08 (1.00, 1.17); p=0.0402; Figure 3(A)). However, the incidence was not significantly different between the naldemedine and magnesium oxide arms of the non-early prescription group (Figure 3(B)). Addition or change of laxatives occurred significantly more frequently in the naldemedine arm than in the magnesium oxide arm in the early prescription group (HR (95% CI), 1.21 (1.12, 1.31); p<0.0001; Figure 4(A)). As expected, dose increase occurred significantly more frequently in the magnesium oxide arm than in the naldemedine arm in the early prescription group (HR (95% CI), 0.04 (0.03, 0.06); p<0.0001; Figure 5(A)). Not only in the naldemedine arm but also in the magnesium oxide arm, addition and change of laxatives were more common than dose increase (Figure 4(A) and Figure 5(A)). The addition or change of laxatives was not significantly different between the naldemedine and magnesium oxide arms of the non-early prescription group (Figure 4(B)). The dose increase occurred significantly more frequently in the magnesium oxide arm than in the naldemedine arm of the non-early prescription group (HR (95% CI), 0.11 (0.06, 0.20); p<0.0001; Figure 5(B)).

**Figure 6:**
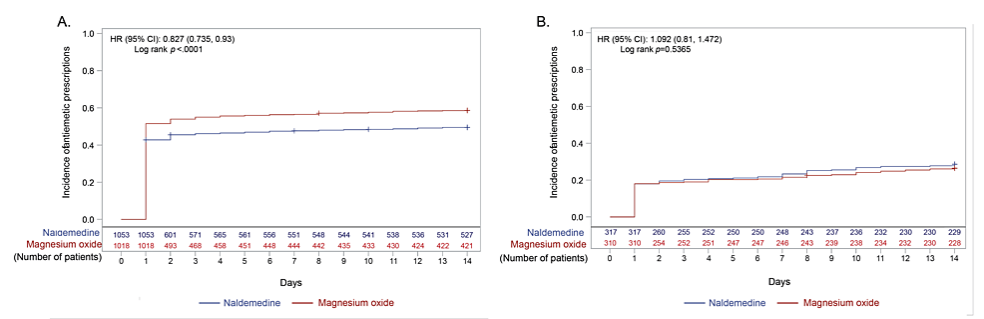
Incidence of antiemetic prescriptions after the start of the opioid prescription in (A) early and (B) non-early prescription groups CI: confidence interval; HR: hazard ratio

Prescriptions of antiemetics were less frequent in the naldemedine arm than in the magnesium oxide arm of the early prescription group (HR (95% CI), 0.83 (0.74, 0.93); p<0.0001; Figure 6(A)). Prescriptions of antiemetics were comparable between the naldemedine and magnesium oxide arms of the non-early prescription group (Figure 6(B)).

The incidence of opioid change was not different between the naldemedine and magnesium oxide arms of the early prescription group (Appendix F(A)), whereas it was significantly higher in the naldemedine arm than in the magnesium oxide arm of the non‑early prescription group (HR (95% CI), 1.25 (1.01, 1.55); p=0.0439; Appendix F(B)). Higher doses of opioids were prescribed in the naldemedine than in the magnesium oxide arm at the index date in the early prescription group (mean (SD) doses in mg; 31.1 (60.4) versus 26.9 (35.6); p=0.012). The trend continued even with increasing doses of opioids at the subsequent time points (Appendix C). In the non-early prescription group, the prescribed dose of opioids tended to increase at later time points, although no difference was seen between the naldemedine and magnesium oxide arms (mean (SD) doses in mg at the index date; 26.8 (30.2) versus 25.7 (26.6); p=0.521). The number of treatments for constipation (stool extraction, enema, etc.) did not differ between the naldemedine and magnesium oxide arms of both the early and non-early prescription groups (Appendix G). Total medical costs for naldemedine were significantly less than that for magnesium oxide in the non-early prescription group (p=0.0488), although there was no statistical difference between the arms in the early prescription group (Appendix E).

## Discussion

This retrospective study highlighted the difference in the prescription patterns of naldemedine and magnesium oxide as first-line medications for the treatment of OIC in patients with cancer pain and the corresponding treatment outcomes.

The naldemedine arm had slightly more composite addition/change/dose increase in the primary endpoint relative to the magnesium oxide arm in the early prescription group. Since dose increase was limited in the naldemedine arm as a function of the dose restriction specified in the approved dosage [23], the high frequency of addition/change of laxatives in the naldemedine arm would mainly contribute to the primary endpoint. About 70-80% of patients received an addition/change of laxatives after opioid initiation in both the naldemedine and magnesium oxide arms in the early prescription group, although to a lesser degree in the magnesium oxide arm, which suggests that multiple laxatives would be required for OIC management. Since naldemedine is an OIC-specific laxative, physicians may intend to prevent OIC development first and, after that, treat the remaining constipation, which may not be OIC (not induced by MOR directly), with other laxatives. There are many factors causing constipation in patients with cancer, and magnesium oxide is possibly effective in treating constipation other than OIC, explaining the fewer prescription changes compared with naldemedine. For instance, it is worth noting that chemotherapy with taxanes within one month of naldemedine initiation is a significant risk for OIC [24] and is significantly associated with non-responsiveness to naldemedine [25]. High body mass index and lack of appropriate MOR antagonists early in the opioid treatment are additional risk factors for OIC [24]. However, it cannot be stated conclusively whether these factors contributed to our study as patients' background treatment was not matched. The higher rate of addition/change/dose increase in the naldemedine arm also suggests that constipation not induced by MOR directly may also be involved. Moreover, the dosage of magnesium oxide might be adjusted depending on the patient's condition before administration, which might decrease the rate of addition/change/dose increase, especially in the early group [10]. In contrast, there was no significant difference in the primary composite endpoint between the naldemedine and magnesium oxide arms in the non-early prescription group. Notably, while just over 10% of the non-early group started naldemedine or magnesium oxide treatment >3 months after starting opioids, the length after starting opioids was matched between naldemedine and magnesium oxide in the non-early group. Furthermore, additions/changes were not significantly different between the naldemedine and magnesium oxide arms. Since OIC would already be developed by the time laxative was prescribed in the non-early prescription group, the physician who prescribed naldemedine may have prescribed it for symptomatic relief rather than prophylactic purposes, eliminating the need for addition/change in treatment. The number of constipation treatments (stool extraction, enema, etc.) was not significantly different between the naldemedine and magnesium oxide arms in both early and non-early prescription groups. It would suggest that the severity of constipation may not be different between the naldemedine and magnesium oxide arms in both early and non-early prescription groups.

In this study, we used the propensity score matching for adjusting patient backgrounds. After matching, there was no difference in the incidence of death in the early prescription group, but there was a difference in the amounts of opioids prescribed at the index date. On the contrary, the incidence of death in the non-early prescription group was higher in the naldemedine arm, but there was no difference in the amounts of opioids prescribed at the start. These differences suggest that patient backgrounds were not fully matched well. The patients with more severe and/or painful cancer who needed opioid initiation might be prescribed naldemedine rather than magnesium oxide, because naldemedine would mechanistically control adverse effects of opioids better than osmotic laxatives. This could explain the higher mortality in the naldemedine arm compared with the magnesium oxide arm. The differences in prescription patterns between the early and non-early prescription groups can be attributed to other factors. Since naldemedine is a single fixed-dose drug, there was no dose increase noted in the naldemedine arm. On the other hand, the frequent dose increase of magnesium oxide can be attributed to the low dose prescription at initiation [10] and limited efficacy in the treatment of OIC [11,26]. Thus, physicians might try to increase the dose as much as the highest tolerable permitted dose. Interestingly, in both the early and non-early prescription groups, additions/changes were more common than dose increases in the magnesium oxide arm. This may reflect the physicians' intention to try different mechanisms when one medication produces an inadequate response. Switching to another medication may also provide a better efficacy and/or safety profile than increasing the dose. Moreover, the usage of a high dose of magnesium oxide would increase the risk of hypermagnesemia [11].

The incidences of antiemetic prescription were made mostly on day 1 in both early and non-early prescription groups. It suggests that most of the antiemetics were prescribed prophylactically. Antiemetic prescriptions were higher in the early prescription group than in the non-early prescription group for both the naldemedine and magnesium oxide arms. The physicians who chose early prescription of laxatives would tend to prescribe antiemetic prophylactically. Moreover, the results of fewer antiemetic prescriptions in the naldemedine arm versus the magnesium oxide arm in the early prescription group may be associated with physicians' perspectives on the mechanistic basis of OINV which is induced by MORs [27]. Naldemedine prevented nausea and vomiting in animals [16] and reduced OINV in the clinical setup [15,19]. This is especially relevant as naldemedine reduced nausea to a greater degree when compared with magnesium oxide during a 12-week administration in a randomized controlled trial [19].

In a retrospective study, it has been reported that naldemedine can reduce hospital costs and hospital stays by reducing the incidence of hyperactive delirium [28]. In the present study, the total medical costs six months after opioid initiation were low in the naldemedine arm compared to the magnesium oxide arm in the non-early prescription group, but not in the early prescription group. Since, as mentioned above, the incidence rates of death, which may reflect the severity of cancer, were different between the naldemedine and magnesium oxide arms in the non-early prescription group, the total medical costs may be affected by the patient's background. On the other hand, no significant difference was seen between the naldemedine and magnesium oxide arms in the early prescription group, suggesting that the frequency of addition/change/dose increase in the naldemedine arm may not have affected the medical cost.

The results should be viewed in light of the study's limitations. First, the database used for this study covered only data from hospitals that had an acute inpatient care system, precluding the inclusion of small hospitals and clinics. Also, this database did not capture data when patients received medications from other hospitals and used over-the-counter medications and did not differentiate between an addition and a change of laxatives. Additionally, the reasons for additions/changes could not be established as they are dependent on the severity and status of constipation. Second, as the data were based on payment-related information, the data do not truly reflect actual use. Third, the clinical symptoms, the severity of constipation, and the general condition of patients, which might affect the selection of laxatives, were not known. Although propensity score matching was conducted, including cancer and opioid details, patient background might still not be matched enough regarding patient condition, including cancer severity and pain, due to the limitation of information in the database. To mitigate this, we defined the start of the opioid prescription as an index date. The initiation of opioid treatment is the root cause of OIC. Having a common index date also allowed us to examine the potential differences between early laxative treatment and non-early treatment in this study. We also acknowledge that the results from the Japanese patient populations may not be directly generalizable to the global population without accounting for local factors. Finally, because naldemedine is approved as a single-dose (0.2 mg/day) prescription [23], dose increment assessments may not be feasible and have limited value as such. Despite the limitations, our study, to the best of our knowledge, is the first study to evaluate treatment status and associated outcomes when naldemedine and magnesium oxide are prescribed as first-line medications for the treatment of OIC in real-world clinical settings in Japan. In summary, while we may not have fully achieved the objectives of this study to compare the outcome after initiating naldemedine and magnesium oxide because of the limitations of the MDV database approach, the analysis provided valuable information on the treatment patterns (timing of addition, change, and dose increase) following naldemedine and magnesium oxide prescriptions.

## Conclusions

In the early prescription group, addition, change, or dose increase of laxatives occurred slightly but more frequently in the naldemedine arm than in the magnesium oxide arm, though antiemetic prescription was less frequent in the naldemedine arm than in the magnesium oxide arm in patients with cancer in Japan. Our findings shed light on the treatment patterns and outcomes in patients prescribed naldemedine and magnesium oxide who have cancer pain and OIC and provide valuable insights into real-world clinical treatment patterns in this patient population. This could, in turn, help develop OIC treatment algorithms to facilitate the right treatment choices. Opioids can be made more accessible to patients suffering from cancer pain if the adverse events can be well tolerated using the appropriate therapy without compromising the pain relief.

## Figures and Tables

**Figure 7:**
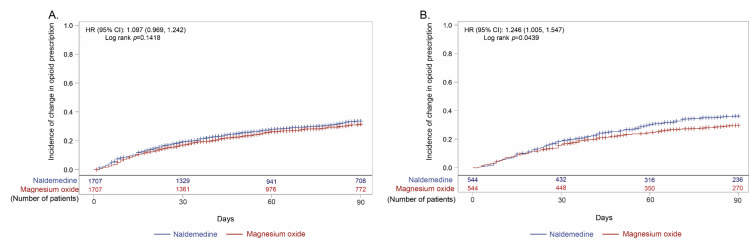
Incidence of change in opioid prescription in (A) early and (B) non-early prescription groups CI: confidence interval; HR: hazard ratio

**Figure 8:**
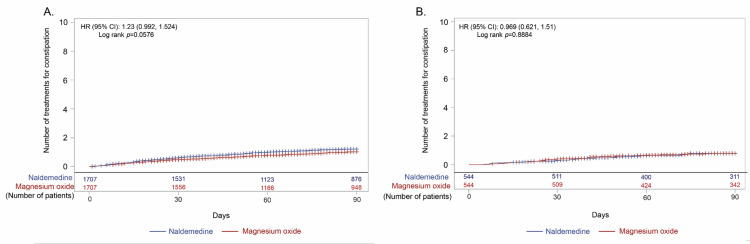
Number of treatments for constipation in (A) early and (B) non-early prescription groups CI: confidence interval; HR: hazard ratio

## Appendices

Appendix A

**Table 3:** List of diagnoses of malignant tumors with their ICD-10 codes ICD-10: International Statistical Classification of Diseases and Related Health Problems

Cancer type	ICD-10 code
Head and neck	C00.x-C14.x and C30.x-C32.x
Upper digestive organs	C15.x-C16.x
Lower digestive organs	C17.x-C21.x
Liver and gallbladder	C22.x-C24.x
Pancreas	C25.x
Respiratory and intrathoracic organs	C33.x-C39.x
Breast	C50.x
Female genital organs	C51.x-C58.x
Male genital organs	C60.x-C63.x
Urinary tract	C64.x-C68.x
Brain and spinal cord including metastasis	C70.x-C72.x and C79.3
Lymphoid and hematopoietic tissue	C81.x-C96.x

Appendix B

**Table 4:** List of opioid rescue prescription drugs

Generic name	Commercial name
Oxycodone hydrochloride hydrate	Oxinorm powder 0.5%
	Okinomu powder 2.5 mg
	Okinomu powder 5 mg
	Okinomu powder 10 mg
	Okinomu powder 20 mg
	Oxycodone tablets 2.5 mg "Daiichi Sankyo"
	Oxycodone tablets 5 mg "Daiichi Sankyo"
	Oxycodone tablets 10 mg "Daiichi Sankyo"
	Oxycodone tablets 20 mg "Daiichi Sankyo"
	Oxycodone tablets 2.5 mg NX "Daiichi Sankyo"
	Oxycodone tablets 5 mg NX "Daiichi Sankyo"
	Oxycodone tablets 10 mg NX "Daiichi Sankyo"
	Oxycodone tablets 20 mg NX "Daiichi Sankyo"
Hydromorphone hydrochloride	Narurapid lock 1 mg
	Narurapid lock 2 mg
	Narurapid lock 4 mg
Fentanyl citrate	Acref oral mucosal absorbent 200 μg
	Acref oral mucosal absorbent 400 μg
	Acref oral mucosal absorbent 600 μg
	Acref oral mucosal absorbent 800 μg
	Ifenbuccal tablet 50 μg
	Ifenbuccal tablet 100 μg
	Ifenbuccal tablet 200 μg
	Ifenbuccal tablet 400 μg
	Ifenbuccal tablet 600 μg
	Ifenbuccal tablet 800 μg
	Abstral sublingual tablet 100 μg
	Abstral sublingual tablet 200 μg
	Abstral sublingual tablet 400 μg
Morphine hydrochloride hydrate	Opso oral solution 5 mg 2.5 mL
	Opso oral solution 10 mg 5 mL
	Ampec suppository 30 mg
	Ampec suppository 10 mg
	Ampec suppository 20 mg
Buprenorphine hydrochloride	Repetan suppository 0.2 mg
	Repetan suppository 0.4 mg
Pentazocine hydrochloride	Sosegon lock 25 mg
	Pertazone tablets 25 25 mg
	Pentazine tablets 25 25 mg
Morphine hydrochloride hydrate	Morphine hydrochloride "Shionogi"
	Morphine hydrochloride hydrate
	Morphine hydrochloride tablet 10 mg
	Morphine hydrochloride tablet "Malpy" 10 mg
	Morphine hydrochloride "Sankyo"
	Morphine hydrochloride hydrate "Daiichi Sankyo" bulk powder
	Morphine hydrochloride tablet 10 mg "DSP"
	Morphine hydrochloride hydrate "Takeda" bulk powder
	Morphine hydrochloride hydrate "Shionogi" bulk powder

Appendix C

**Table 5:** Prescribed opioid doses with naldemedine and magnesium oxide treatments All data are reported as mg. Opioid doses are expressed as morphine equivalent doses [29,8,30,31]. SD: standard deviation

	Naldemedine		Magnesium oxide		P-value
	n	Average (SD)	n	Average (SD)	
Early					
Starting date	1707	31.1 (60.4)	1707	26.9 (35.6)	0.012
Three days	1703	33.4 (69.3)	1706	27.8 (34.0)	0.003
One week	1701	35.8 (54.9)	1706	30.5 (38.8)	0.001
Two weeks	1695	39.9 (55.4)	1699	34.0 (42.9)	0.001
One month	1627	47.8 (62.3)	1635	41.0 (53.5)	0.001
Two months	1205	50.6 (68.8)	1226	41.2 (45.1)	<0.0001
Three months	942	50.9 (72.5)	1001	41.2 (43.4)	<0.001
Non-early					
Starting date	544	26.8 (30.2)	544	25.7 (26.6)	0.521
Three days	544	28.2 (33.2)	544	26.3 (27.1)	0.311
One week	544	30.4 (33.4)	544	28.2 (27.4)	0.238
Two weeks	544	35.5 (43.3)	543	31.8 (31.1)	0.111
One month	524	43.8 (53.3)	527	38.5 (39.8)	0.069
Two months	415	47.3 (50.1)	441	44.6 (68.7)	0.515
Three months	318	50.3 (62.3)	352	42.6 (51.8)	0.082

Appendix D

**Table 6:** Propensity score prediction formula in early and non-early prescription groups ^a^The variable "overlap of malignant tumor" becomes 1 when all variables of other cancer types are 0 (linear combination) and is used as a reference for calculating the estimated value of the prediction formula, so it is not output to the prediction formula. ^a^c-statistic, 0.604; Hosmer-Lemeshow test, p=0.9808. ^b^The variable "overlap of malignant tumor" becomes 1 when all other cancer variables are 0 (linear combination) and is not output to the prediction formula because it is used as a reference for calculating the prediction formula estimated value. ^b^The variable "period until the start of prescription of laxatives period exceeding 3 months" becomes 1 when the variable for the period until the start of prescription of other laxatives is 0 (linear combination) and is used as a reference for calculating the estimated value of the prediction formula. No output in the prediction formula. ^b^c-statistic, 0.607; Hosmer-Lemeshow test, p=0.7901. CI: confidence interval

	Estimate	Odds ratio	95% CI	P-value
Early prescription^a^				
Slice	-1.041	-	-	0.016
Age	-0.001	0.999	0.994, 1.003	0.530
Sex	0.018	1.018	0.904, 1.147	0.765
Cancer type				
Head and neck	-0.139	0.870	0.602, 1.258	0.460
Gastrointestinal tract	0.050	1.051	0.868, 1.274	0.609
Liver, bile, pancreas	0.294	1.342	1.118, 1.612	0.002
Lung	0.070	1.073	0.891, 1.291	0.458
Mammary gland	-0.173	0.841	0.592, 1.196	0.336
Gynecologic cancer	0.531	1.701	1.208, 2.395	0.002
Carcinoma of the renal/urinary system	0.097	1.102	0.827, 1.469	0.509
Blood	-0.580	0.560	0.381, 0.824	0.003
Other	0.020	1.020	0.798, 1.303	0.876
Prescription classification	-0.085	0.918	0.820, 1.028	0.139
Opioid oxycodone hydrochloride hydrate	-0.110	0.896	0.423, 1.894	0.773
Opioid tapentadol hydrochloride	0.286	1.332	0.610, 2.908	0.472
Opioid hydromorphone hydrochloride	1.076	2.932	1.381, 6.222	0.005
Opioid morphine	-0.035	0.965	0.441, 2.115	0.930
Opioid fentanyl	-0.098	0.907	0.432, 1.905	0.797
Peritoneal seeding	0.332	1.393	1.144, 1.698	0.001
Non-early prescription^b^				
Slice	-2.440	-	-	0.011
Age	0.002	1.002	0.994, 1.01	0.633
Sex	0.177	1.194	0.971, 1.467	0.093
Cancer type				
Head and neck	-0.069	0.933	0.442, 1.971	0.856
Gastrointestinal tract	0.267	1.306	0.980, 1.741	0.069
Liver, bile, pancreas	0.153	1.165	0.847, 1.603	0.348
Lung	-0.184	0.832	0.586, 1.182	0.305
Mammary gland	-0.793	0.453	0.220, 0.933	0.032
Gynecologic cancer	0.695	2.003	1.176, 3.413	0.011
Carcinoma of the renal/urinary system	0.227	1.254	0.792, 1.986	0.334
Blood	-0.658	0.518	0.270, 0.994	0.048
Other	0.250	1.285	0.821, 2.009	0.272
Prescription classification	-0.147	0.863	0.692, 1.077	0.193
Time until the start of laxative prescription				
~2 weeks	0.226	1.254	0.911, 1.727	0.165
One month	0.156	1.169	0.834, 1.638	0.365
Two months	-0.072	0.930	0.629, 1.376	0.718
Three months	-0.162	0.850	0.515, 1.405	0.527
Opioid oxycodone hydrochloride hydrate	0.673	1.960	0.339, 11.353	0.453
Opioid tapentadol hydrochloride	1.295	3.650	0.610, 21.855	0.156
Opioid hydromorphone hydrochloride	1.312	3.713	0.629, 21.898	0.147
Opioid morphine	0.853	2.346	0.378, 14.571	0.360
Opioid fentanyl	0.468	1.597	0.275, 9.273	0.602
Peritoneal seeding	-0.072	0.930	0.669, 1.293	0.667

Appendix E

**Table 7:** Total medical costs for patients in naldemedine and magnesium oxide treatment arms after six months of treatment The values are reported in Japanese yen. Q1: first quartile; Q3: third quartile; SD: standard deviation

Group	Naldemedine			Magnesium oxide			P-value
	n	Average (SD)	Median (Q1, Q3)	n	Average (SD)	Median (Q1, Q3)	
Early at six months	1707	2520861 (1984033)	2031485 (1093279, 3481167)	1707	2630484 (3953000)	2054632 (1054191, 3497669)	0.306
Non-early at six months	544	2310472 (1703936)	1921214 (1040981, 3179736)	544	2534608 (2029672)	1972040 (1091122, 3384527)	0.049

Appendix F

Appendix G
